# Manejo Invasivo versus Conservador de Pacientes com IAMSSST Com Idade ≥ 75 Anos

**DOI:** 10.36660/abc.20220658

**Published:** 2023-05-18

**Authors:** Mengjin Hu, Xiaosong Li, Yuejin Yang

**Affiliations:** 1 Fuwai Hospital State Key Laboratory of Cardiovascular Disease Beijing China Fuwai Hospital State Key Laboratory of Cardiovascular Disease, Beijing – China; 2 Xuanwu Hospital Capital Medical University Beijing China Xuanwu Hospital, Capital Medical University, Beijing – China

**Keywords:** Idoso, Infarto do Miocárdio, Intervenção Coronária Percutânea, Manejo Invasivo

## Abstract

**Fundamento:**

A eficiência do manejo invasivo em pacientes mais velhos (≥75 anos) com infarto do miocárdio sem supradesnivelamento do segmento ST (IAMSSST) permanece ambígua.

**Objetivos:**

Avaliar a eficiência do tratamento invasivo em pacientes idosos com IAMSSST com base em metanálise e análise sequencial de estudo (TSA).

**Métodos:**

Ensaios clínicos randomizados relevantes (ECR) e estudos observacionais foram incluídos. Os resultados primários foram morte por todas as causas, infarto do miocárdio, acidente vascular cerebral e hemorragia grave. O odd ratio agrupado (OR) e o intervalo de confiança de 95% (IC) foram calculados. P<0,05 foi considerado estatisticamente significativo.

**Resultados:**

Cinco ECRs e 22 estudos observacionais com 1.017.374 pacientes foram incluídos.Com base nos resultados de ECR e TSA, o manejo invasivo foi associado a menores riscos de infarto do miocárdio (OR: 0,51; 95% IC: 0,40-0,65; I2=0%), eventos cardiovasculares adversos maiores (MACE; OR: 0,61; 95% IC: 0,49-0,77; I2=27,0%) e revascularização (OR: 0,29; 95% IC: 0,15-0,55; I2=5,3%) em comparação com o tratamento conservador. A combinação de resultados de ECRs e estudos observacionais com ajuste multivariável mostrou riscos consistentemente menores de morte por todas as causas (OR: 0,57; IC 95%: 0,50-0,64; I2 = 86,4%), infarto do miocárdio (OR: 0,63; IC 95%: 0,56 -0,71; I2=0%), acidente vascular cerebral (OR: 0,59; 95% IC: 0,51-0,69; I2=0%) e MACE (OR: 0,64; 95% IC: 0,54-0,76; I2=43,4%). O melhor prognóstico associado ao manejo invasivo também foi observado em cenários do mundo real. No entanto, para pacientes com idade ≥85 anos, o manejo invasivo pode aumentar o risco de sangramento maior (OR: 2,68; IC 95%: 1,12-6,42; I2=0%).

**Conclusões:**

O manejo invasivo foi associado a menores riscos de infarto do miocárdio, MACE e revascularização em pacientes idosos com IAMSSST,no entanto, pode aumentar o risco de sangramento maior em pacientes com idade ≥85 anos.

## Introdução

A idade avançada é um preditor crucial para resultados adversos em pacientes com síndrome coronariana aguda (SCA), pois foram observados maiores riscos de mortalidade a curto e longo prazo em pacientes mais velhos em comparação com os mais jovens.^1^ As diretrizes atuais enfatizam o tratamento intervencionista intensivo e precoce em pacientes com SCA, particularmente aqueles com riscos mais elevados de eventos de curto prazo.^2 , 3^ Os pacientes idosos representam um subgrupo conhecido por estar em risco aumentado e podem se beneficiar da revascularização na mesma extensão que os pacientes mais jovens.^4^ No entanto, dados do banco de dados National Inpatient Sample nos EUA sugeriram que a taxa de angiografia coronária invasiva no infarto do miocárdio sem supradesnivelamento do segmento ST (IAMSSST) diminuiu com a idade, com apenas 38% dos pacientes com 81 anos ou mais recebem angiografia coronária invasiva, em comparação com 78% dos pacientes com 60 anos ou menos,^5^ o que pode ser explicado pelas preocupações sobre um risco potencial aumentado de complicações após procedimentos de revascularização.^6^

Devido ao rápido crescimento da população idosa, a Organização Mundial da Saúde prevê que as mortes causadas por doenças coronarianas aumentarão em 120-137% durante as próximas duas décadas.^7^ Como é a principal causa de morte globalmente,^8^ determinar uma estratégia eficiente para o tratamento de pacientes idosos com IAMSSST é essencial.No entanto, os pacientes idosos estão sub-representados em ensaios clínicos randomizados (ECR), pois a idade média dos pacientes inscritos é inferior a 75 anos nos ECRs. Portanto, a generalização e a tradução dos resultados do ECR para pacientes mais velhos são limitadas.Enquanto isso,o número de ECRs com foco no manejo invasivo em pacientes idosos (≥75 anos) com IAMSSST é limitado e pode ter pouca potência para os resultados de interesse. Consequentemente, o manejo de pacientes idosos com IAMSSST continua sendo uma questão complicada.

Na presente metanálise, nosso objetivo foi avaliar os eventos clínicos relacionados ao manejo invasivo em pacientes com IAMST com idade ≥ 75 anos com base em ECRs e análise sequencial de ensaios (TSA).^9^A TSA ajuda a determinar se um ECR pode ser encerrado precocemente quando um valor de p é suficientemente pequeno para mostrar o efeito antecipado ou a futilidade.^10^ Enquanto isso, estudos observacionais também foram incluídos para nos ajudar a entender os cenários do mundo real na prática clínica.

## Métodos

### Desenho e seleção do estudo

ECRs e estudos observacionais comparando invasivo (intervenção coronária percutânea [ICP]/enxerto de revascularização do miocárdio [CRM]) versus tratamento conservador em pacientes idosos (≥75 anos) com IAMSSST e relatando resultados clínicos foram considerados. Estudos com foco em pacientes com angina instável ou IAMCSST foram excluídos. Estudos relevantes foram pesquisados PubMed, Web of Science, Biblioteca Cochrane, ClinicalTrials.gov e Google Scholar usando as seguintes palavras-chave: idosos, septuagenários, octogenários, nonagenários, infarto do miocárdio, infarto do miocárdio sem supradesnivelamento do segmento ST, IAMSSST, invasivo, agressivo, intervenção coronária percutânea, ICP, cirurgia de revascularização miocárdica, CRM, angioplastia, seletiva, conservadora, terapia médica, terapia medicamentosa desde a publicação até 9 de maio de 2022. Dois investigadores revisaram independentemente os títulos, resumos e estudos para determinar se eles atendiam à inclusão critério. Esta metanálise foi realizada de acordo com a declaração Preferred Reporting Items for Systematic Review and Meta-Analysis,^11^ e foi registrada no International Prospective Register of Systematic Reviews (CRD42022301170).

## Resultados

Os desfechos primários foram morte por todas as causas, infarto do miocárdio (IM), acidente vascular cerebral e sangramento maior, de acordo com a definição de cada estudo individual. O desfecho secundário incluiu eventos cardiovasculares adversos maiores (MACE), morte cardíaca, revascularização e reinternação.

### Análise TSA

Na análise TSA, os ECRs são incluídos em ordem cronológica e a análise é realizada de forma repetitiva e cumulativa após a realização de novos ECRs. TSA também fornece um nível de significância ajustado para controlar os erros Tipo I e II.^12^ TSA ajuda a determinar se um ECR pode ser encerrado antecipadamente quando um valor p é suficientemente pequeno para mostrar o efeito antecipado ou futilidade.^10^ Quando a curva Z cumulativa cruza o limites de monitoramento sequencial de teste, indica que a análise é válida para benefício. A TSA foi conduzida pelo software TSA, versão 0.9 beta (Copenhagen Trial Unit, Copenhagen, Dinamarca).

### Análise estatística

Dados brutos e não ajustados de ECRs incluídos e estudos observacionais foram extraídos. O odd ratio agrupado (OR) e o intervalo de confiança de 95% (IC) foram calculados usando modelos de efeito aleatório (DerSimonian e Laird). Além disso, devido ao número limitado de ECRs, resultados combinados de ECRs e estudos observacionais com ajuste multivariável também foram calculados. A análise de subgrupo foi realizada de acordo com a idade dos pacientes (≥75, ≥80 e ≥85). A análise de meta-regressão foi realizada para explorar ainda mais a heterogeneidade dos efeitos do tratamento, estratificando por idade e percentual de revascularização. Além disso, uma análise de exclusão também foi realizada para avaliar se um único estudo influenciou os resultados agrupados. O viés de publicação foi avaliado por inspeção visual de gráficos de funil e Teste de Begg. A heterogeneidade entre os estudos foi avaliada usando a estatística^12 , 13^ com I^2^ <25%, 25%-75% e >75% considerados baixo, moderado e alto, respectivamente. P<0,05 foi considerado estatisticamente significativo. Todas as análises estatísticas foram realizadas usando Stata 16 SE (StataCorp, College Station, TX).

## Resultados

### Características iniciais dos estudos incluídos

**Figura 1:**
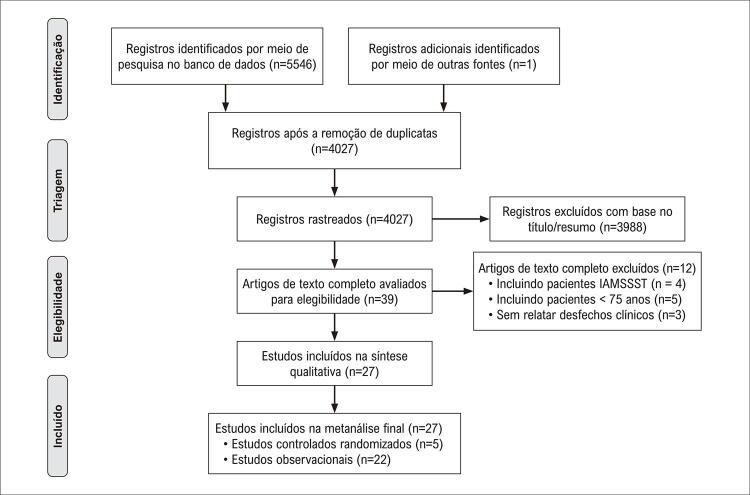
– Diagrama PRISMA para Inclusão no Estudo. IAMCSST: infarto do miocárdio com supradesnivelamento do segmento ST.

Conforme mostrado na Figura 1 , de 5.546 estudos potencialmente relevantes, cinco ECRs^14 - 18^e 22 estudos observacionais^19 - 40^preencheram os critérios de inclusão. Um total de 178.860 (17,6%) pacientes foram tratados de forma invasiva, enquanto 838.514 (82,4%) foram tratados de forma conservadora. As principais características dos estudos e pacientes incluídos são apresentadas na Tabela 1 .

**Tabela 1:** – Características iniciais dos estudos incluídos

Estudo, ano	Design de estudo	Número de pacientes	Definição de MACE	Anos de idade)	Masc. (%)	Hipertensão (%)	Hiperlipidemia (%)	Diabetes (%)	FEVE (%)	Insuficiência cardíaca	Insuficiência renal (%)	Acesso radial (%)	Antagonista da GP IIb/IIIa (%)	Tempo de acompanhamento
TÁTICAS–TIMI ^14 , 18^ 2004	ECR, multicêntrico	139/139	Morte cardíaca, IM ou necessidade de revascularização coronária não planejada	N / D	N / D	N / D	N / D	N / D	N / D	N / D	N / D	N / D	N / D	6 meses
Registro ACOS ^19^ 2007	Prospectivo, multicêntrico, observacional	1005/931	Morte e IM	78,7/82,2	51,7/41,4	79,2/74,8	62,3/49,7	33,5/39,4	N / D	N / D	4.5/9.2	N / D	N / D	12 meses
GRACE, ^20^ 2008	Prospectivo, multicêntrico, observacional	620/2390	Morte, IM e AVC	85	48	73	39	26	N / D	27	11	N / D	15	6 meses
LOURENÇO, ^21^ 2010	Prospectivo, de centro único, observacional	91/216	Morte, morte cardiovascular, infarto do miocárdio, readmissão por angina instável e ICP não programada	79,8/81,4	63,7/50,9	80,2/74,9	60,7/62,5	26,4/30,8	N / D	3,8/10,2	N / D	N / D	39,6/30,1	18 meses
FIR, ^15^ 2012	ECR, multicêntrico	437/402	IM e infarto do miocárdio	76	63,5	39,0	15.7	14.8	N / D	N / D	N / D	N / D	N / D	5 anos
FAST-MI, ^22^ 2012	Prospectivo, multicêntrico, observacional	412/246	N / D	N / D	N / D	N / D	N / D	N / D	N / D	N / D	N / D	N / D	N / D	3 anos
Italian Elderly ACS, ^16^ 2012	ECR, multicêntrico,	154/159	Morte por todas as causas, IM, AVC, e repetir internação	81,8/81,8	49/51	92/85	44/50	38/41	49/48	10/8.9	N / D	71/78	17/6	1 ano
ACSIS, ^23^ 2013	Prospectivo, multicêntrico, observacional	192/158	N / D	83	54	81	N / D	36	N / D	29	N / D	N / D	6	1 ano
PL-ACS, ^24^ 2013	Prospectivo, multicêntrico, observacional	3288/10419	N / D	82/83	47,5/37,2	78,6/71,0	36,3/32,6	30,9/30,5	N / D	N / D	N / D	N / D	4,6/0,1	2 anos
Kolte, ^25^ 2013	Retrospectivo, multicêntrico, observacional	161640/ 806902	N / D	83,9/86,2	48.2/41	72/62,5	51,9/33,7	28.0/29.1	N / D	34,8/55	16,8/22,8	N / D	N / D	Hospitalização
MONICA/KORA MI, ^26^ 2016	Prospectivo, multicêntrico, observacional	360/286	N / D	78/80	55,8/46/5	90,6/85,0	47,8/33,6	40,0/50,0	N / D	N / D	24,7/36,4	N / D	24,7/4,9	28 dias
Conti, ^27^ 2016	Prospectivo, de centro único, observacional	301/152	Morte e IM	80	52,8/34,2	91,7/90,8	53,5/38,8	41.2/42.1	N / D	N / D	N / D	N / D	N / D	1 ano
HUMIR, ^28^ 2016	Prospectivo, multicêntrico, observacional	654	N / D	N / D	N / D	N / D	N / D	N / D	N / D	N / D	N / D	N / D	N / D	1 ano
Estudo After Eighty, ^17^ 2016	ECR, multicêntrico	229/228	IM, necessidade de revascularização urgente, AVC e morte	84,7/84,9	55/44	57/61	N / D	20/14	N / D	N / D	N / D	90	N / D	3 anos
AMI-OPTIMA, ^40^ 2018	Subanálise de ECR, multicêntrico	548/610	N / D	85,6	47,6	78,3	53.4	31.2	N / D	N / D	25.3	N / D	N / D	no Hospital
Liu, ^29^ 2018	Retrospectivo, de centro único, observacional	319	N / D	N / D	N / D	N / D	N / D	N / D	N / D	N / D	N / D	N / D	N / D	32,3 meses
LONGEVO-SCA, ^30^ 2018	Prospectivo, multicêntrico, observacional	407/124	Morte cardíaca, IM ou necessidade de revascularização coronária não planejada	83,6/86,7	64,7/50,0	84.0/87.1	N / D	39,0/42,7	53,2/53,7	14,3/29,0	N / D	84,5	N / D	6 meses
SWEDE HEART, ^31^ 2018	Prospectivo, multicêntrico, observacional	4158/9696	N / D	84/86	57/46	63/63	N / D	N / D	N / D	N / D	N / D	N / D	N / D	5 anos
Kvakkestad, ^32^ 2019	Prospectivo, centro único, observacional	1200/864	N / D	80,4/86,4	60,6/39,7	50,1/48,4	N / D	20,7/19,0	N / D	N / D	N / D	N / D	N / D	7 anos
CAMI, ^33^ 2019	Prospectivo, multicêntrico, observacional	551/1900	Morte por todas as causas, IM e AVC	80,37	54,6	65,8	6.0	25,0	53,41	10.8	N / D	N / D	10.8	No Hospital
Sui, ^34^ 2019	Retrospectivo, de centro único, observacional	139/93	N / D	83,4/84,8	52,5/57,0	66,9/68,8	93,5/90,3	40,3/34,4	51,9/45,3	N / D	23,7/36,6	N / D	N / D	4 anos
Gonçalves, ^35^ 2020	Retrospectivo, multicêntrico, observacional	237/87	N / D	87/87	54,0/60,9	86,4/90,7	58,8/59,5	29,6/43,7	52/49	N / D	14.1/9.4	64,5/80,7	N / D	1 ano
Hirlekar, ^18^ 2020	ECR, multicêntrico	93/93	IM, revascularização urgente, mortalidade por todas as causas, AVC e hospitalização recorrente	84/84	50,5/59,1	59,1/63,4	22,6/17,2	17,2/21,5	55/54	10,8/8,6	63,4/75,3	N / D	N / D	1 ano
SENIOR-NSTEMI, ^36^ 2020	Prospectivo, multicêntrico, observacional	655/845	N / D	85,3/86,9	60/50	62/54	42/30	26/24	N / D	17/24	7/8	N / D	N / D	4,8 anos
Nguyen, ^37^ 2020	Prospectivo, multicêntrico, observacional	42/78	Mortalidade por todas as causas, IM e AVC	84.1/85.2	59,5/44,9	97,6/85,9	N / D	21.4/23.1	N / D	21,4/48,7	N / D	N / D	N / D	3 meses
Phan, ^38^ 2020	Retrospectivo, de centro único, observacional	890/543	N / D	83,3/83,3	64,6/67,4	91.9/94.1	93,0/93,6	49,1/56,9	51/45	44,5/64,8	58,1/63,9	N / D	N / D	2,6 anos
Kunniardy, ^39^ 2021	Retrospectivo, de centro único, observacional	99/953	N / D	N / D	N / D	N / D	N / D	N / D	N / D	N / D	N / D	N / D	N / D	1,3 anos

Todos os estudos adotaram 5% de significância estatística. Os dados foram relatados como manejo invasivo/conservador. CRM: cirurgia de revascularização miocárdica; MACE: eventos cardiovasculares adversos maiores; FEVE: fração de ejeção do ventrículo esquerdo; IM: infarto do miocárdio; ND: não disponível; ICP: intervenção coronária percutânea; ECR: ensaio controlado randomizado; AVC: acidente vascular cerebral.

### Resultados clínicos baseados em ECRs

**Figura 2:**
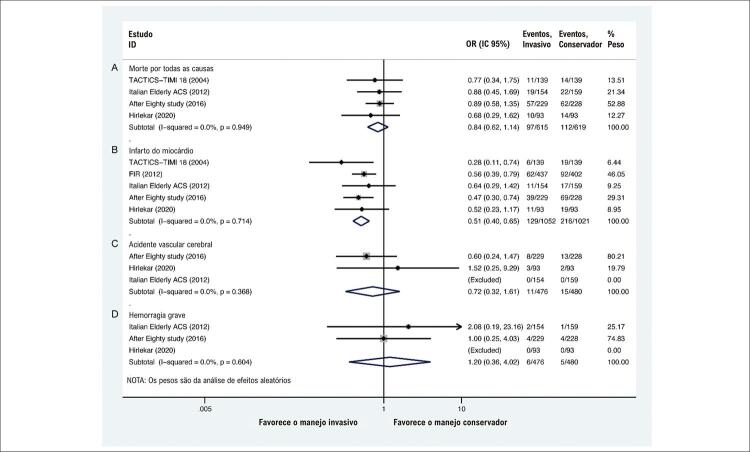
– Comparações de resultados primários com base em ensaios controlados randomizados. A) morte por todas as causas; B) infarto do miocárdio; C) acidente vascular cerebral; D) hemorragia grave.

**Figura 3:**
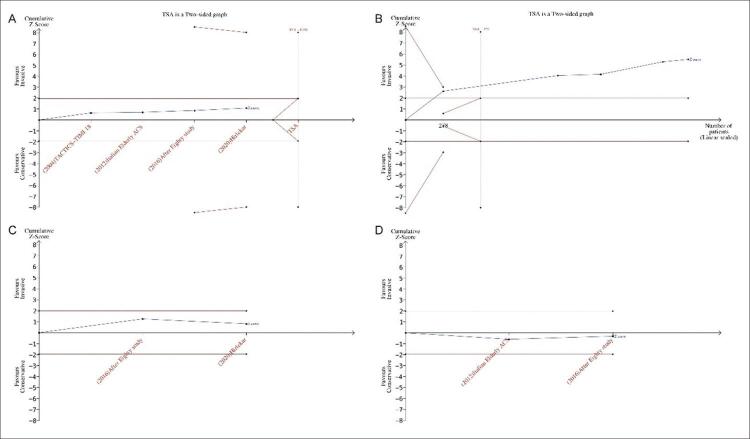
– Resultados TSA para resultados primários. A) morte por todas as causas; B) infarto do miocárdio; C) acidente vascular cerebral; D) hemorragia grave. IM: infarto do miocárdio; TSA: análise sequencial do ensaio. A linha azul representa o Z-score cumulativo da metanálise. As linhas transversais vermelhas representam os limites estatísticos convencionais de p = 0,05. As linhas vermelhas inclinadas para dentro representam os limites de monitoramento sequencial de teste. As linhas vermelhas inclinadas para fora representam o limite de futilidade. As linhas verticais vermelhas representam o tamanho da informação necessária ajustada à diversidade.

Era óbvio que o manejo invasivo estava associado a menores riscos de infarto do miocárdio (OR: 0,51; IC 95%: 0,40-0,65; I^2^=0%; Figura 2B), sem influência significativa na morte por todas as causas (Figura 2A), AVC (acidente vascular cerebral) (Figura 2C) ou sangramento maior (Figura 2D). Para todas as causas de morte (Figura 3A), a curva Z cumulativa não ultrapassou os limites estatísticos convencionais, os limites de monitoramento sequencial do ensaio ou o tamanho de informação necessário ajustado à diversidade, indicando que não foi obtida informação suficiente. A curva Z cumulativa cruzou os limites de monitoramento sequencial do teste para benefício em IM (Figura 3B), indicando que informações suficientes foram obtidas. Para AVC (Figura 3C) e sangramento maior (Figura 3D), os resultados da TSA foram ignorados devido à pouca informação utilizada.

Quanto aos desfechos secundários, o manejo invasivo foi associado a menores riscos de MACE (Material suplementar 1A) e revascularização (Material suplementar 1C) em comparação com o manejo conservador, sem diferenças significativas em morte cardíaca (Material suplementar 1B) ou readmissão (Material suplementar 1D). A curva Z cumulativa ultrapassou os limites de monitoramento sequencial do estudo para benefício em MACE (Material suplementar 2A) e revascularização (Material suplementar 2C). Em contraste, não foram obtidas informações suficientes para morte cardíaca (Material suplementar 2B) ou reinternação (Material suplementar 2D).

### Combinando resultados de ECRs e estudos observacionais com ajuste multivariável

**Figura 4:**
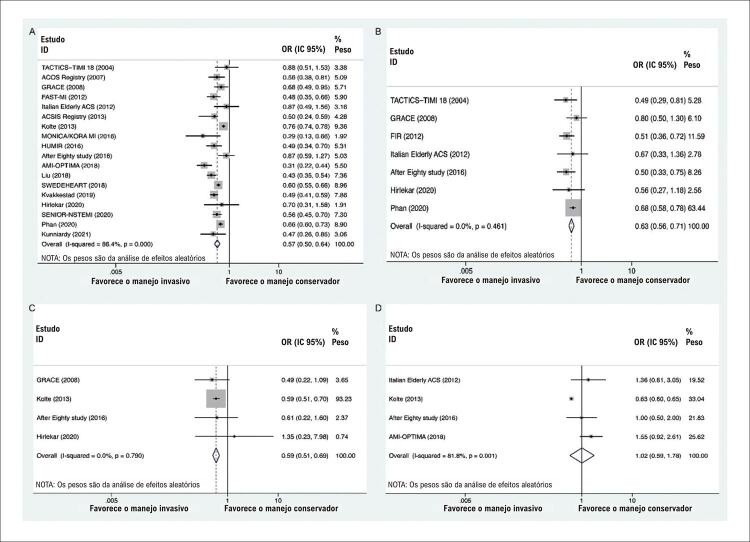
– Combinando resultados primários de Ensaios Randomizados Controlados Ensaios e Estudos Observacionais com Ajuste Multivariável. A) morte por todas as causas; B) infarto do miocárdio; C) acidente vascular cerebral; D) hemorragia grave.

Como os resultados da TSA revelaram que informações suficientes foram obtidas apenas para IM, MACE e revascularização, mas não para outros desfechos, os resultados de ECRs e estudos observacionais com ajuste multivariável também foram calculados para aumentar o tamanho da amostra e atenuar o viés o máximo possível. Os resultados indicaram que o manejo invasivo foi consistentemente associado a menores riscos de morte por todas as causas (OR: 0,57; IC 95%: 0,50-0,64; I^2^=86,4%; Figura 4A), IM (OR: 0,63; IC 95%: 0,56 -0,71; I^2^=0%; Figura 4B) e acidente vascular cerebral (OR: 0,59; IC 95%: 0,51-0,69; I^2^=0%; Figura 4C) em relação ao tratamento conservador, sem aumentar o risco de sangramento maior (Figura 4D). Além disso, o manejo invasivo pode reduzir o risco de MACE ( Material suplementar 3A) sem influências significativas na morte cardíaca ( Material suplementar 3B), revascularização ( Material suplementar 3C) ou readmissão ( Material suplementar 3D).

### O cenário do mundo real baseado em estudos observacionais

**Figura 5:**
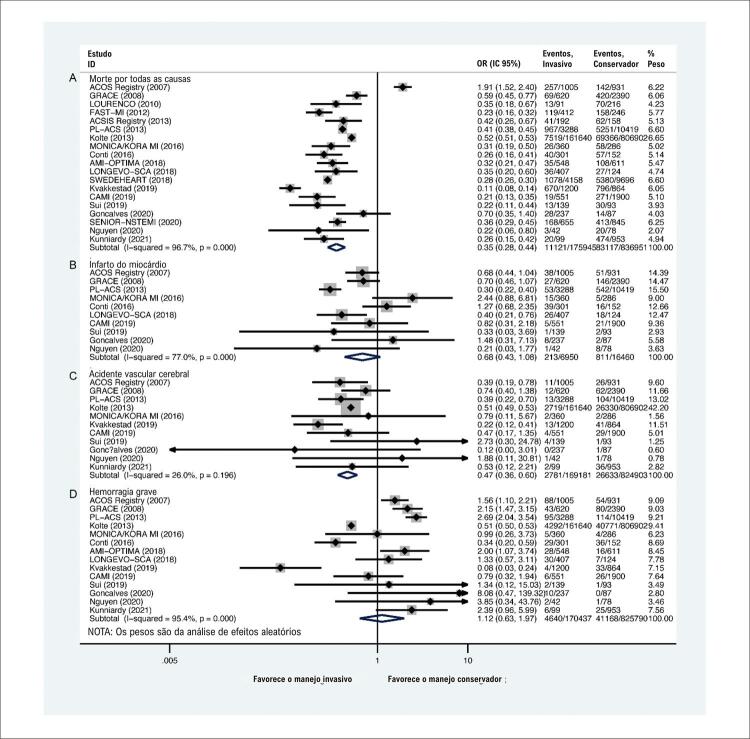
– Comparações de Resultados Primários com Base em Estudos Observacionais do Mundo Real. A) morte por todas as causas; B) infarto do miocárdio; C) acidente vascular cerebral; D) hemorragia grave.

Os resultados de estudos observacionais revelaram que o manejo invasivo pode diminuir os riscos de morte por todas as causas (OR: 0,35; 95% IC: 0,28-0,44; I^2^=96,7%; Figura 5A) e acidente vascular cerebral (OR: 0,47; 95% IC: 0,36 -0,60; I^2^=26,0%; Figura 5C), sem repercussão em IM (Figura 5B) e sangramento maior (Figura 5D). Além disso, o manejo invasivo pode diminuir os riscos de MACE (OR: 0,41; IC 95%: 0,32-0,53; I^2^=64,2%; Material suplementar 4A) e morte cardíaca (OR: 0,32; IC 95%: 0,23-0,47; I^2^= 0%;Figura suplementar 4B).

### Viés de publicação, análises de sensibilidade, análises de meta-regressão e análises de subgrupo

As distribuições do gráfico de funil ( Material suplementar 5) e os testes de Begg ( Material suplementar 6) não revelaram nenhum viés de publicação para todos os resultados.Nas análises de sensibilidade de exclusão, os resultados permaneceram consistentes com a análise primária ( Material suplementar 7). As análises de meta-regressão sobre a idade e a porcentagem de tratamento invasivo não revelaram efeitos nos resultados clínicos entre o tratamento invasivo e conservador (Tabela complementar 1). A análise de subgrupo de morte por todas as causas de acordo com a idade dos pacientes sugeriu que os benefícios em morte por todas as causas ( Material suplementar 8A), IM ( Material suplementar 8B) e acidente vascular cerebral ( Material suplementar 8C) foram consistentes, exceto para pacientes mais velhos com mais de 85 anos, nos quais o manejo invasivo pode aumentar o risco de sangramento maior (OR: 2,68; IC 95%: 1,12-6,42; I^2^=0%; Material suplementar 8D) sem benefício nos demais parâmetros avaliados. Para desfechos secundários, o manejo invasivo foi associado a um menor risco de MACE ( Material suplementar 9A), independentemente da idade, com risco reduzido de morte cardíaca ( Material suplementar 9B) e revascularização ( Material suplementar 9C) em pacientes com mais de 80 anos.

## Discussão

Nesta metanálise e TSA, nossos achados podem ser resumidos da seguinte forma: (1) o manejo invasivo diminuiu os riscos de infarto do miocárdio, MACE e revascularização com evidências sólidas baseadas em resultados de ECRs e TSA; (2) nenhuma diferença significativa em morte por todas as causas, acidente vascular cerebral, sangramento maior, morte cardíaca e reinternação entre o tratamento invasivo e conservador em ECRs pode ser explicada pelo número limitado de pacientes incluídos de acordo com os resultados da TSA; (3) agrupar os resultados de ECRs e estudos observacionais com ajuste multivariado revelou que o manejo invasivo foi associado a riscos menores de morte por todas as causas, infarto do miocárdio, acidente vascular cerebral e MACE em relação ao manejo conservador; (4) O cenário do mundo real de estudos observacionais também sugeriu que o manejo invasivo pode diminuir os riscos de morte por todas as causas, acidente vascular cerebral, MACE e morte cardíaca; (5) para pacientes idosos com idade ≥85 anos, o manejo invasivo pode aumentar o risco de sangramento maior.

Por muito tempo, o tratamento de pacientes idosos com IAMSSST tem sido uma questão desafiadora e complicada, pois esses pacientes mais velhos têm maior probabilidade do que seus colegas mais jovens de apresentar sintomas atípicos, como ausência de dor torácica na SCA.^41^ Além disso, a idade avançada per se é considerada um fator de risco independente para morbidade e mortalidade precoces após a apresentação de IAMSSST.^42^ Além disso, o pior desfecho associado a pacientes idosos é influenciado não apenas por doença arterial coronariana extensa, mas também por doenças mais complexascomorbidades,^43^ como doença de calcificação coronariana multiarterial complexa, anatomia vascular tortuosa, função ventricular prejudicada, perfil de maior risco e comorbidade substancial.^44^ Todas as razões acima contribuíram para a incerteza sobre a relação risco-benefício do manejo invasivo.

Dados do mundo real mostraram que pacientes mais velhos com IAMSSST acompanhados de múltiplas comorbidades eram menos propensos a receber tratamento invasivo, possivelmente devido a uma relação risco-benefício desfavorável percebida. Nossa metanálise mostra que pacientes que não receberam manejo invasivo eram mais propensos a ter insuficiência cardíaca ou insuficiência renal. Talvez a preocupação com a nefropatia induzida por contraste os tenha impedido de receber manejo invasivo. No estudo After Eighty,^17^ 457 pacientes com IAMSSST com idade ≥80 anos foram designados aleatoriamente para uma estratégia invasiva (n=229) ou uma estratégia conservadora (n=228). Durante um acompanhamento médio de 1,53 anos, o desfecho primário definido como um composto de infarto do miocárdio, necessidade de revascularização urgente, acidente vascular cerebral e morte ocorreu com menos frequência no grupo invasivo em comparação com o grupo conservador (40,6% vs. 61,4%; risco ratio [HR]: 0,53; IC 95%: 0,41–0,69; p=0,0001), devido principalmente aos riscos reduzidos de IM (HR: 0,52; IC 95%: 0,35–0,76; p=0,0010) e revascularização urgente (HR: 0,19; IC 95%: 0,07–0,52; p=0,0010). Na metanálise realizada por Abusnina et al., eles compararam a eficiência de uma estratégia invasiva em pacientes com IAMSSST com mais de 80 anos. Um total de três ECRs e 893 pacientes foram incluídos.^45^ Em comparação com a estratégia conservadora,de acordo com nossos resultados de TSA, os resultados não significativos em morte por todas as causas, acidente vascular cerebral e sangramento maior entre o manejo invasivo e conservador em ECRs podem ser explicados pelo número limitado de pacientes incluídos e pela falta de poder para os resultados de interesse. Portanto, mais estudos são necessários para validar o efeito de umestratégia invasiva em pacientes com IAMSSST. Além disso, considerando o número limitado de ECRs, os resultados agrupados de ECRs e estudos observacionais com ajuste multivariado também foram conduzidos como análises complementares. A inclusão dos últimos estudos relevantes pode tornar os resultados mais convincentes. Além disso, os critérios de inclusão e exclusão foram rígidos nos ECRs, o que pode limitar a generalidade dos resultados à prática do mundo real; portanto, a análise de subgrupo baseada apenas em estudos observacionais também foi realizada. Todas as análises acima indicaram consistentemente que a estratégia invasiva foi superior ao tratamento conservador. No entanto, um risco aumentado de sangramento maior foi observado em pacientes com idade ≥85 anos, o que sugere cautela ao considerar o tratamento invasivo em pacientes muito idosos.^46^

Na prática do mundo real, aproximadamente metade dos pacientes com IAMSSST com estenose significativa não foi submetida a ICP.^19^ As razões podem ser explicadas por doença de pequenos vasos inelegível para tratamento invasivo, doença arterial coronariana grave (por exemplo, multiarterial/tronco principal esquerdo) combinada com grave doença periférica e doença arterial coronariana grave após CRM inelegível para refazer cirurgia e ICP. No estudo conduzido por Phan et al.,^38^ foram relatados os dois motivos mais comuns para o manejo conservador: 1. baixa candidatura ao manejo invasivo devido à fragilidade, anatomia coronária abaixo do ideal, comorbidades médicas ou outros motivos a critério do médico ( 38,9%); 2. doença arterial coronariana obstrutiva significativa com alta relação risco-benefício, o que favorece uma tentativa de terapia médica primeiro (36,3%).

Os dados de nossa metanálise revelaram uma associação positiva entre uma estratégia invasiva e melhores resultados, mas o benefício de uma estratégia invasiva pode ser diluído pelo peso da idade, com risco aumentado de sangramento maior em pacientes com idade ≥85 anos. Devido ao número limitado de ECRs, estudos mais extensos e ECRs são obrigatórios para esclarecer o papel do manejo invasivo em pacientes idosos com IAMSSST. O estudo SENIOR-RITA (NCT03052036) foi desenvolvido para determinar se uma estratégia invasiva reduz a morte cardiovascular ou infarto do miocárdio em pacientes com IAMSSST com idade ≥75 anos quando comparada com uma estratégia de tratamento conservador. No entanto, estima-se que o ensaio seja concluído até 2029.

### Limitação

Algumas limitações devem ser reconhecidas em nossa metanálise. Primeiro, o manejo invasivo foi realizado de forma mista ICP e/ou CRM. No entanto, análises de subgrupos com base em ECRs recentes demonstraram resultados comparáveis após ICP ou CRM em pacientes idosos, sendo a ICP preferida para pacientes frágeis com maiores riscos de eventos periprocedimentos, enquanto

a CRM é melhor para alcançar a revascularização completa.^47 , 48^ Em segundo lugar, MACE é comumente usados em nossos estudos incluídos, mas seus componentes e combinações diferem. Portanto, MACE foi considerado como um desfecho secundário em vez de primário em nossa metanálise.

## Conclusão

Entre os pacientes mais velhos (≥75 anos) com IAMSSST, o manejo invasivo pode diminuir os riscos de IM, MACE e revascularização com evidências sólidas baseadas em resultados de ECRs e TSA. A combinação de resultados de ECRs e estudos observacionais com ajuste multivariável indicou consistentemente que o manejo invasivo foi melhor para melhorar o prognóstico. No entanto, para pacientes muito idosos com idade ≥85 anos, o manejo invasivo pode aumentar o risco de sangramento maior, o que deve chamar nossa atenção.

## *Material suplementar

Para informação adicional, por favor,clique aqui


