# Supplementary material for: Manejo Invasivo versus Conservador de Pacientes com IAMSSST Com Idade ≥ 75 Anos
Source: Arq Bras Cardiol. 2023 May 18;120(6):e20220658. [Article in Portuguese] doi: 10.36660/abc.20220658 (PMC10484564; doi:10.36660/abc.20220658)
Supplement: Supplementary file 1 [file 2022-0658_AO_SupplementaryMaterial.pdf]

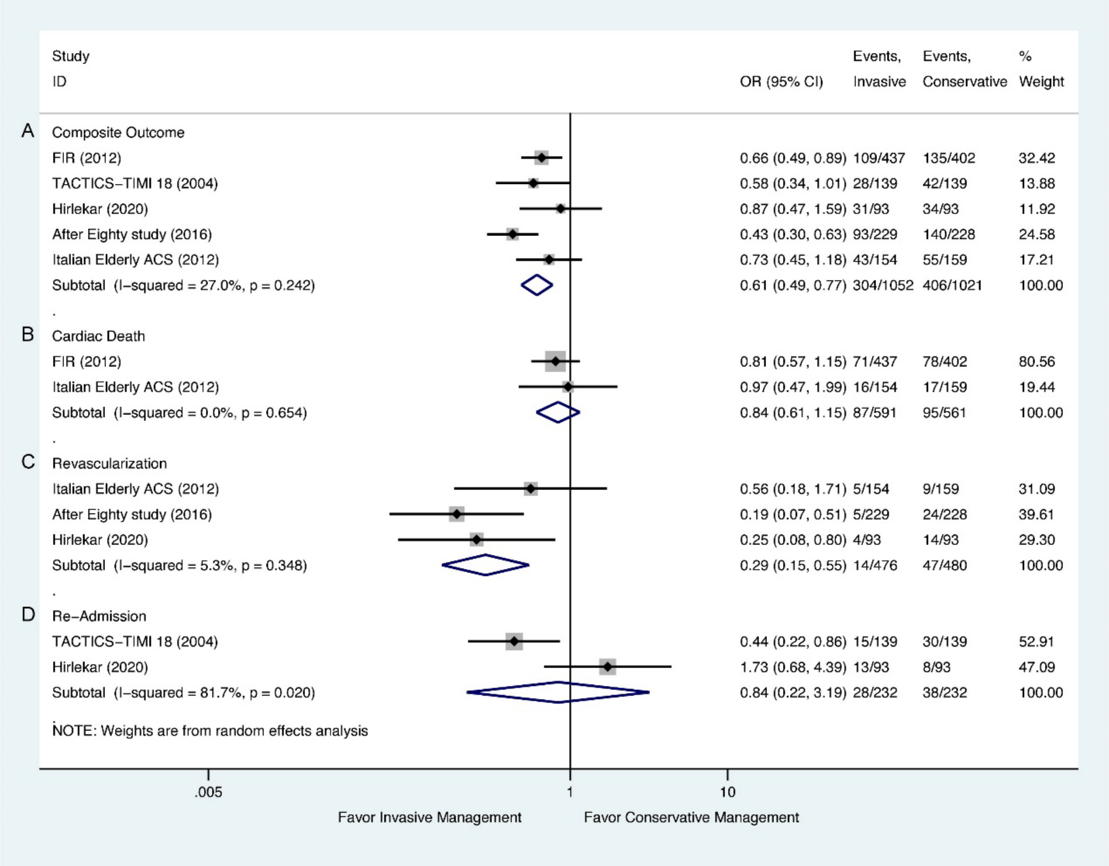

Supplementary Figure 1 Comparisons of Secondary Outcomes Based on Randomized Controlled Trials. A. MACE; B. cardiac death; C. re-admission.

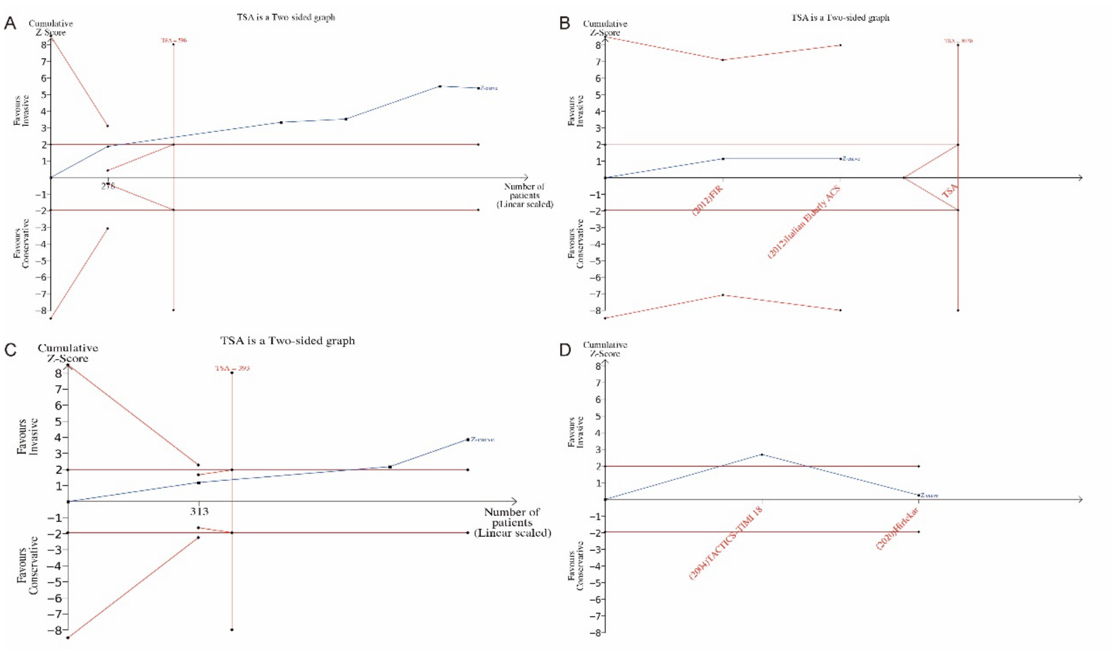

Supplementary Figure 2 TSA Results for Secondary Outcomes

A. MACE; B. cardiac death; C. revascularization; D. re-admission. TSA: sequential analysis. The blue line represents the cumulative Z-score of analysis. The red transverse lines represent the conventional statistical of p = 0.05. The red inward sloping lines represent the trial sequential boundaries. The red outward sloping lines represent the futility boundary. vertical lines represent the diversity-adjusted required information size.

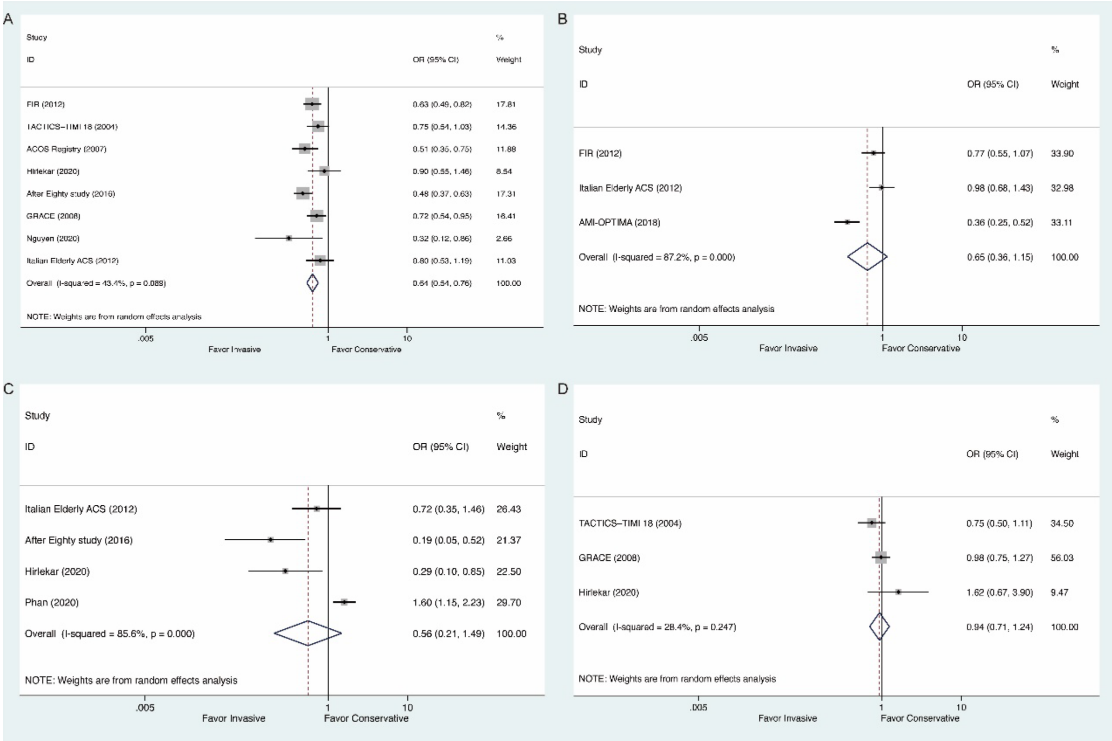

Supplementary Figure 3 Pooling Secondary Outcomes from Randomized Controlled Trials and Observational Studies with Multivariable Adjustment

A. MACE; B. cardiac death; C. revascularization; D. re-admission.

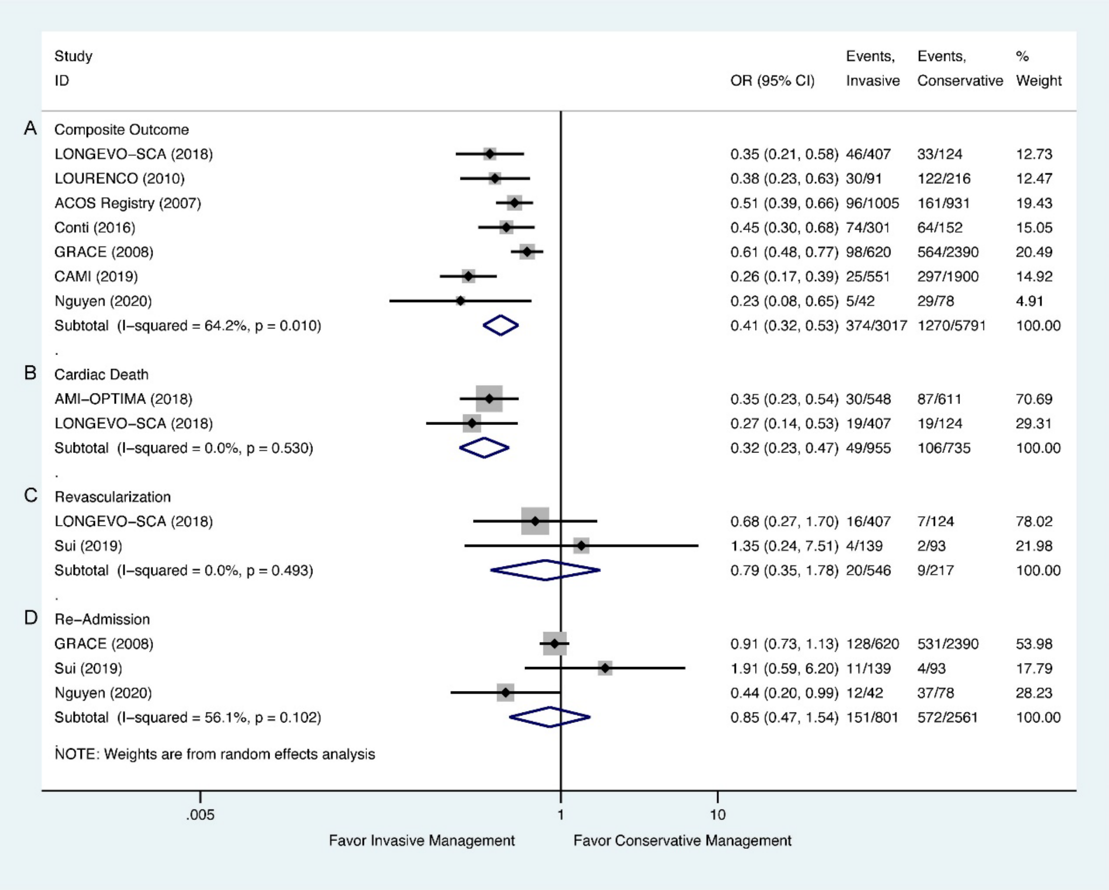

Supplementary Figure 4 Comparisons of Secondary Outcomes Based on Real-World Observational Studies.

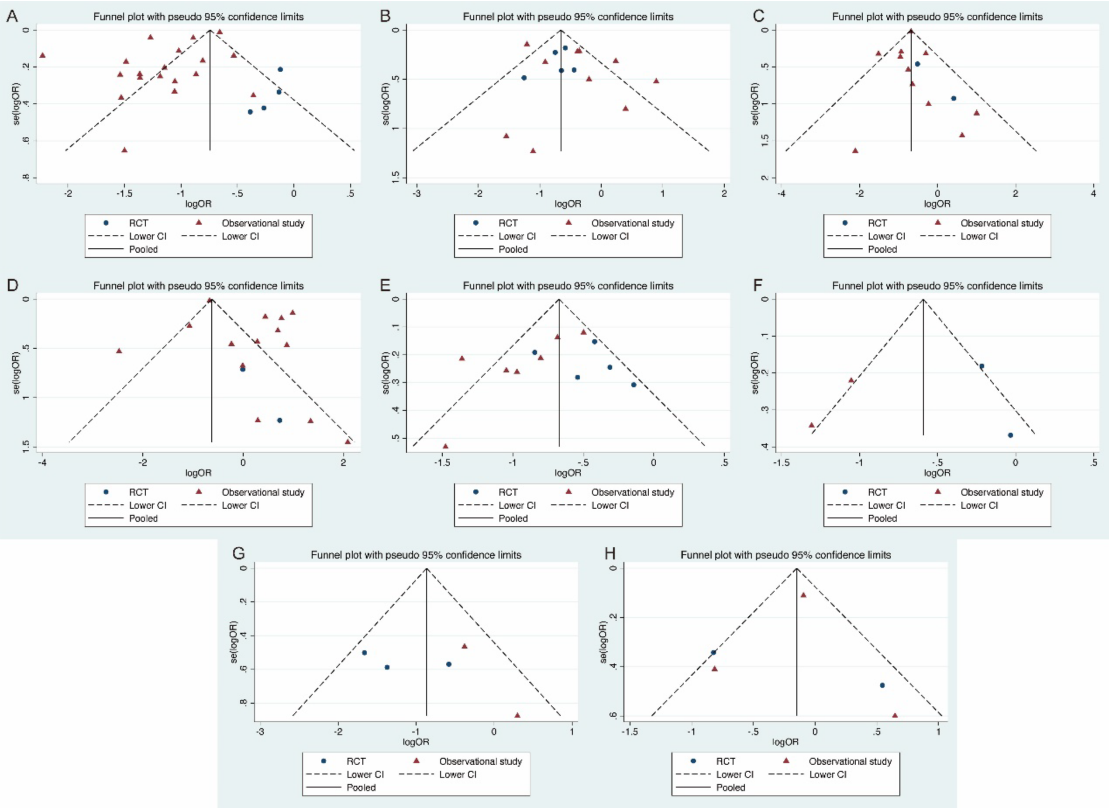

Supplementary Figure 5 Funnel Plot of Publication Bias for Primary and Secondary Outcomes. A. all-cause death; B. myocardial infarction; C. major bleeding; E. MACE; F. cardiac death; G. revascularization; H. re-

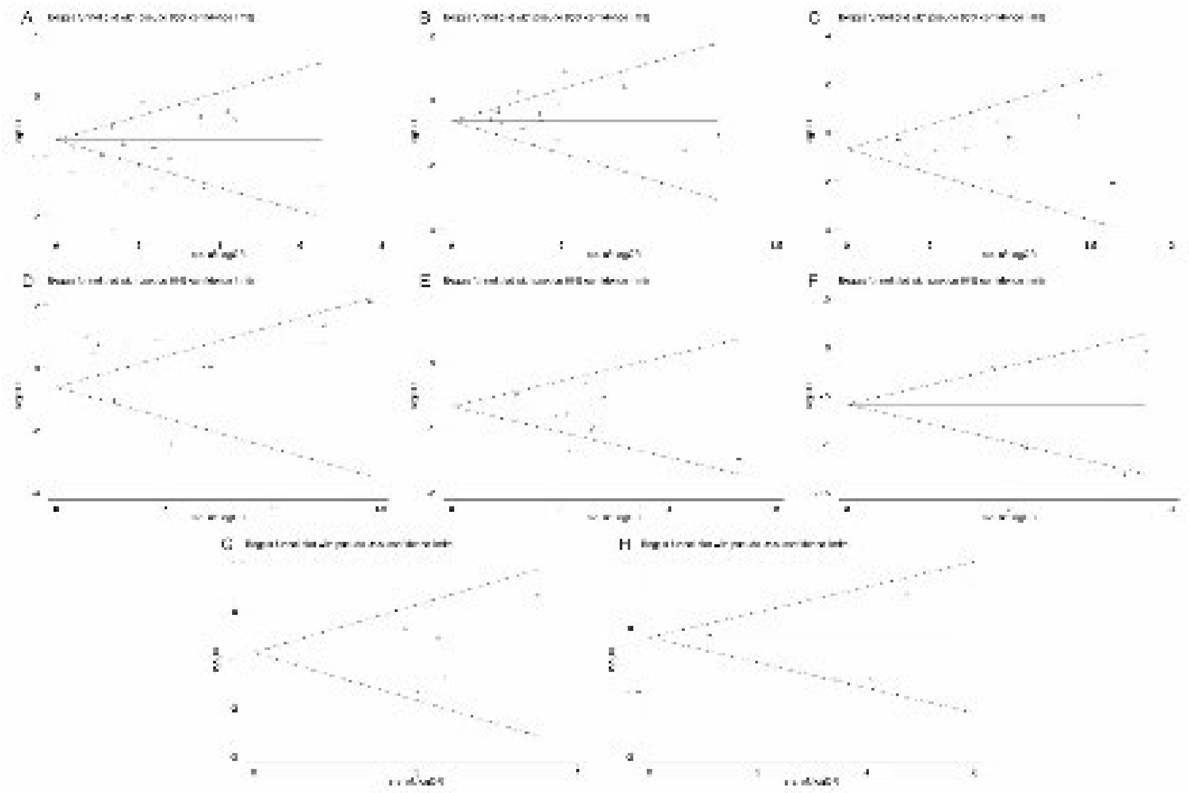

Supplementary Figure 6 Begg's Test for Primary and Secondary Outcomes

A. all-cause death; B. myocardial infarction; C. stroke; D. major bleeding; F. cardiac death; G. revascularization; H. re-admission.

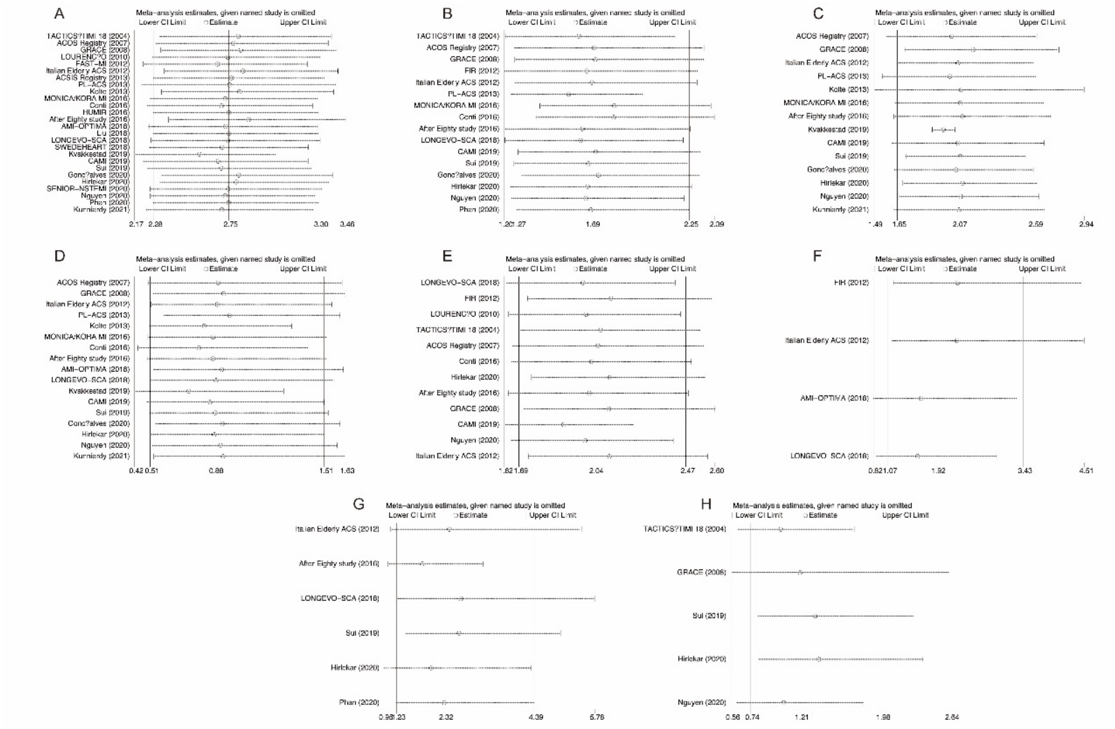

Supplementary Figure 7 Sensitivity Analyses for Primary and Secondary Outcomes. A. all-cause death; B. myocardial infarction; C. stroke; D. E. MACE; F. cardiac death; G. revascularization; H. re-admission.

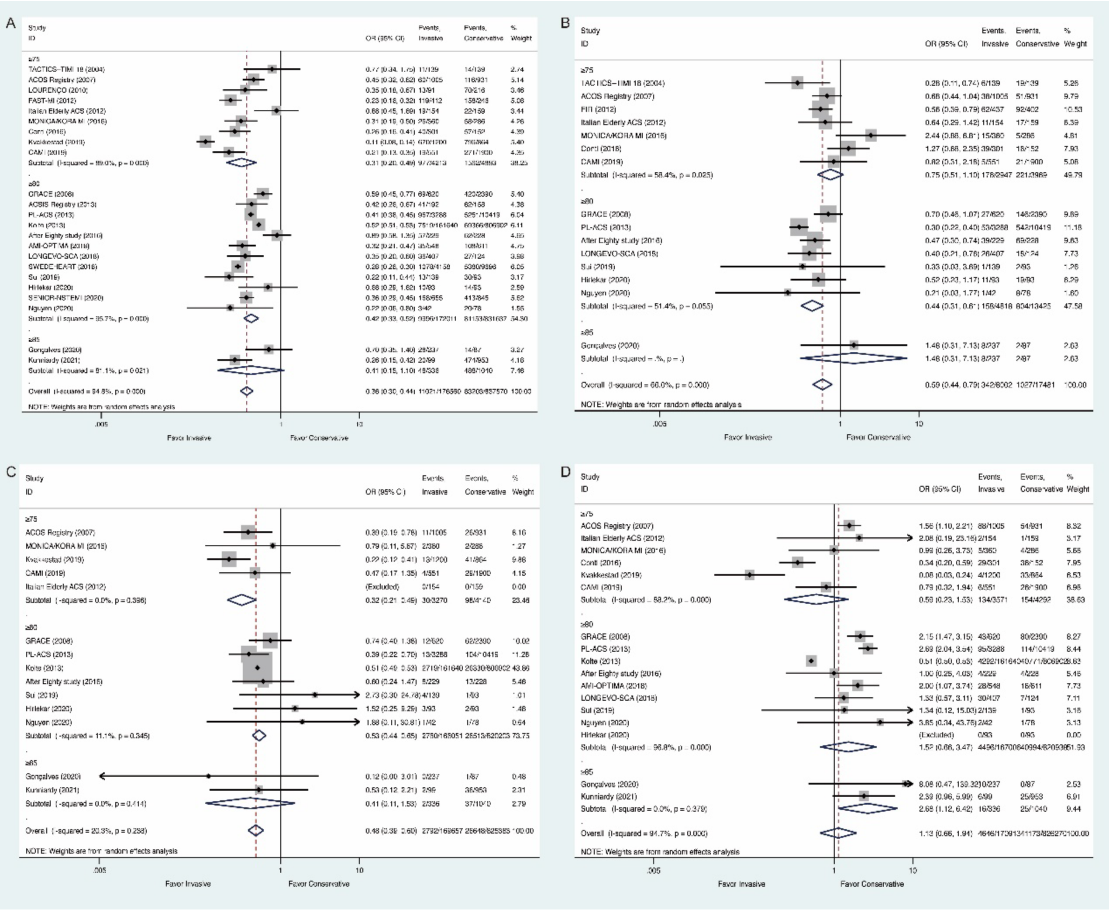

Supplementary Figure 8 Subgroup Analysis of Primary Outcomes According Patients' Age. A. all-cause death; B. myocardial infarction; C. stroke; bleeding.

	Age		Percentage, %	
	Coef.	P	Coef.	P
Composite	-0.012	0.783	0.003	0.662
Death	0.057	0.282	-0.002	0.802
Cardiac death	-0.098	0.243	-0.027	0.423
Myocardial infarction	-0.053	0.316	0.008	0.350
Major bleeding	0.113	0.350	-0.011	0.375
Readmission	-0.867	0.297	0.013	0.634
Revascularization	-0.208	0.580	0.033	0.294
Stroke	0.061	0.387	-0.008	0.196

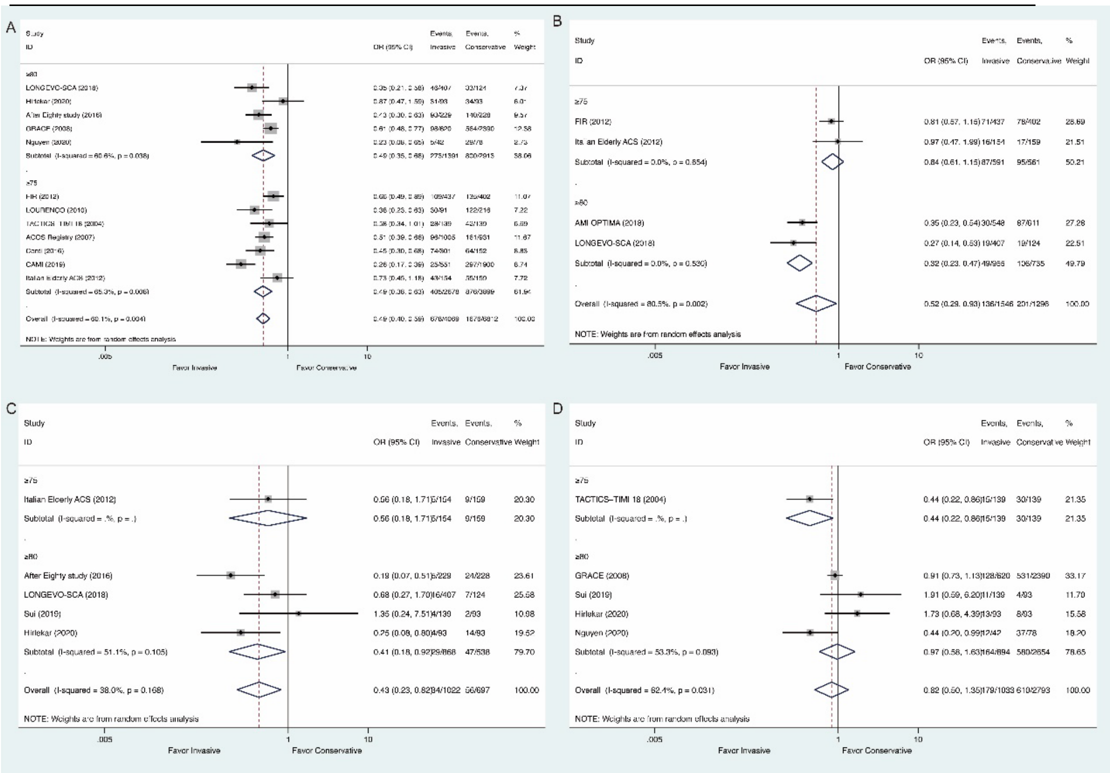

Supplementary Figure 9 Subgroup Analysis of Secondary Outcomes According to Patients' Age. A. MACE; B. cardiac death; C. revascularization; D. re-

Supplementary Table 1. Meta-regression Analyses on Age and the Percentage of Invasive

Management

	Age		Percentage, %	
	Coef.	P	Coef.	P
Composite	-0.012	0.783	0.003	0.662
Death	0.057	0.282	-0.002	0.802
Cardiac death	-0.098	0.243	-0.027	0.423
Myocardial infarction	-0.053	0.316	0.008	0.350
Major bleeding	0.113	0.350	-0.011	0.375
Readmission	-0.867	0.297	0.013	0.634
Revascularization	-0.208	0.580	0.033	0.294
Stroke	0.061	0.387	-0.008	0.196
