# Supplementary material for: Manejo Invasivo versus Conservador de Pacientes com IAMSSST Com Idade ≥ 75 Anos
Source: Arq Bras Cardiol. 2023 May 18;120(6):e20220658. [Article in Portuguese] doi: 10.36660/abc.20220658 (PMC10484564; doi:10.36660/abc.20220658)
Supplement: Translation

# Translation: Invasive Versus Conservative Management of NSTEMI Patients Aged ≥ 75 Years

**Authors:** Mengjin Hu, Xiaosong Li, Yuejin Yang

**Affiliations:** 1 China Fuwai Hospital State Key Laboratory of Cardiovascular Disease, Beijing – China; 2 China Xuanwu Hospital, Capital Medical University, Beijing – China

**Keywords:** Aged, Myocardial Infarction, Percutaneous Coronary Intervention, Conservative Treatment

## Abstract

**Background:**

The efficiency of invasive management in older patients (≥75 years) with non-ST-segment elevation myocardial infarction (NSTEMI) remains ambiguous.

**Objectives:**

To assess the efficiency of invasive management in older patients with NSTEMI based on meta-analysis and trial sequential analysis (TSA).

**Methods:**

Relevant randomized controlled trials (RCT) and observational studies were included. The primary outcomes were all-cause death, myocardial infarction, stroke, and major bleeding. Pooled odd ratio (OR) and 95% confidence interval (CI) were calculated. P <0.05 was considered statistically significant.

**Results:**

Five RCTs and 22 observational studies with 1017374 patients were included. Based on RCT and TSA results, invasive management was associated with lower risks of myocardial infarction (OR: 0.51; 95% CI: 0.40-0.65; I^2^=0%), major adverse cardiovascular events (MACE; OR: 0.61; 95% CI: 0.49-0.77; I^2^=27.0%), and revascularization (OR: 0.29; 95% CI: 0.15-0.55; I^2^=5.3%) compared with conservative management. Pooling results from RCTs and observational studies with multivariable adjustment showed consistently lower risks of all-cause death (OR: 0.57; 95% CI: 0.50-0.64; I^2^=86.4%), myocardial infarction (OR: 0.63; 95% CI: 0.56-0.71; I^2^=0%), stroke (OR: 0.59; 95% CI: 0.51-0.69; I^2^=0%), and MACE (OR: 0.64; 95% CI: 0.54-0.76; I^2^=43.4%). The better prognosis associated with invasive management was also observed in real-world scenarios. However, for patients aged ≥85 years, invasive management may increase the risk of major bleeding (OR: 2.68; 95% CI: 1.12-6.42; I^2^=0%).

**Conclusions:**

Invasive management was associated with lower risks of myocardial infarction, MACE, and revascularization in older patients with NSTEMI, yet it may increase the risk of major bleeding in patients aged ≥85 years.

## Introduction

Older age is a crucial predictor for adverse outcomes in patients with acute coronary syndrome (ACS), as higher risks of short- and long-term mortality were observed in older patients compared to younger counterparts.^1^Current guidelines emphasize intensive and early interventional treatment in ACS patients, particularly those with higher risks of short-term events.^2 , 3^Elderly patients represent a subgroup known to be at increased risk, and they may benefit from revascularization to the same extent as younger patients.^4^However, data from the National Inpatient Sample database in the USA suggested that the rate of invasive coronary angiography in non-ST-segment elevation myocardial infarction (NSTEMI) declined with age, with only 38% of patients who are aged 81 years or older receiving invasive coronary angiography, as compared with 78% of patients who are aged 60 years or younger,^5^which may be explained by the worries about a potential increased risk of complications following revascularization procedures.^6^

Because of the rapid growth of the elderly population, the World Health Organization predicts that the deaths caused by coronary heart disease will increase by 120-137% during the next two decades.^7^As the leading cause of death globally,^8^determining an efficient strategy for treating elderly patients with NSTEMI is essential. However, elderly patients are underrepresented in randomized controlled trials (RCT), as the average age of enrolled patients is younger than 75 years in RCTs. Therefore, the generalizability and translation of RCT results to older patients are limited. Meanwhile, the number of RCTs focusing on invasive management in older patients (≥75 years) with NSTEMI is limited and may be underpowered for the outcomes of interest. Consequently, the management of elderly patients with NSTEMI remains a tricky issue.

In the present meta-analysis, we aimed to assess the clinical events related to invasive management in NSTEMI patients aged ≥ 75 years based on RCTs and trial sequential analysis (TSA).^9^TSA helps determine whether an RCT could be terminated early when a p value is sufficiently small to show the anticipated effect or futility.^10^Meanwhile, observational studies were also included to help us understand real-world scenarios in clinical practice.

## Methods

### Study design and selection

RCTs and observational studies comparing invasive (percutaneous coronary intervention [PCI]/coronary artery bypass grafting [CABG]) versus conservative management in older patients (≥75 years) with NSTEMI and reporting clinical outcomes were considered. Studies focusing on patients with unstable angina or STEMI were excluded. Relevant studies were searched through PubMed, Web of Science, the Cochrane Library, ClinicalTrials.gov, and Google Scholar using the following keywords: elderly, older, septuagenarians, octogenarians, nonagenarians, myocardial infarction, non-ST-segment elevation myocardial infarction, NSTEMI, invasive, aggressive, percutaneous coronary intervention, PCI, coronary artery bypass grafting, CABG, angioplasty, selective, conservative, medical therapy, drug therapy from publication to May 9th, 2022. Two investigators independently reviewed the titles, abstracts, and studies to determine whether they met the inclusion criteria. This meta-analysis was performed according to the Preferred Reporting Items for Systematic Review and Meta-Analysis statement,^11^and it has been registered in the International Prospective Register of Systematic Reviews (CRD42022301170).

### Outcomes

The primary outcomes were all-cause death, myocardial infarction (MI), stroke, and major bleeding, according to the definition of per individual study. The secondary outcome included major adverse cardiovascular events (MACE), cardiac death, revascularization, and re-admission.

### TSA analysis

In TSA analysis, RCTs are included in chronological order, and analysis is performed repetitively and cumulatively after new RCTs are conducted. TSA also provides an adjusted significance level for controlling Type I and II errors.^12^TSA helps determine whether an RCT could be terminated early when a p value is sufficiently small to show the anticipated effect or futility.^10^When the cumulative Z-curve crosses the trial sequential monitoring boundaries, it indicates the analysis is valid for benefit. TSA was conducted by TSA software, version 0.9 beta (Copenhagen Trial Unit, Copenhagen, Denmark).

### Statistical analysis

Raw, unadjusted data from included RCTs and observational studies were extracted. The pooled odd ratio (OR) and 95% confidence interval (CI) were calculated by using random-effect (DerSimonian and Laird) models. Moreover, due to the limited number of RCTs, pooling results from RCTs and observational studies with multivariable adjustment were also calculated. Subgroup analysis was performed according to patients’ age (≥75, ≥80, and ≥85). Meta-regression analysis was performed to explore the heterogeneity of treatment effects further, stratifying by age and percentage of revascularization. Moreover, a leave-one-out analysis was also conducted to assess whether a single study influenced the pooled results. Publication bias was evaluated by visual inspection of funnel plots and Begg’s test. Heterogeneity across studies was assessed using the I^2^statistic,^12 , 13^with I^2^<25%, 25%-75%, and >75% considered low, moderate, and high, respectively. P <0.05 was considered statistically significant. All statistical analyses were performed using Stata 16 SE (StataCorp, College Station, TX).

## Results

### Baseline characteristics of included studies

As shown in Figure 1 , of 5546 potentially relevant studies, five RCTs^14 - 18^and 22 observational studies^19 - 40^met the inclusion criteria. A total of 178860 (17.6%) patients were managed invasively, whereas 838514 (82.4%) were managed conservatively. The major characteristics of the included studies and patients are shown in Table 1 .

**Figure 1:** – PRISMA Diagram for Study Inclusion. STEMI: ST-segment elevation myocardial infarction.

**Table 1:** – Baseline characteristics of included studies

Study, year	Study design	Number of patients	Definition of MACE	Age (years)	Male (%)	Hypertension (%)	Hyperlipidemia (%)	Diabetes (%)	LVEF (%)	Heart failure	Renal failure (%)	Radial access (%)	GP IIb/IIIa antagonist (%)	Follow-up time
TACTICS–TIMI^14 , 18^2004	RCT, multicenter	139/139	cardiac death, MI or need for unplanned coronary revascularisation	NA	NA	NA	NA	NA	NA	NA	NA	NA	NA	6 months
ACOS Registry, ^19^ 2007	prospective, multicenter, observational	1005/931	death and MI	78.7/82.2	51.7/41.4	79.2/74.8	62.3/49.7	33.5/39.4	NA	NA	4.5/9.2	NA	NA	12 months
GRACE, ^20^ 2008	prospective, multicenter, observational	620/2390	death, MI, and stroke	85	48	73	39	26	NA	27	11	NA	15	6 months
LOURENÇO, ^21^ 2010	prospective, single-center, observational	91/216	death, cardiovascular death, MI, re-admission for unstable angina, and unscheduled PCI	79.8/81.4	63.7/50.9	80.2/74.9	60.7/62.5	26.4/30.8	NA	3.8/10.2	NA	NA	39.6/30.1	18 months
FIR, ^15^ 2012	RCT, multicenter	437/402	cardiovascular death and MI	76	63.5	39.0	15.7	14.8	NA	NA	NA	NA	NA	5 years
FAST-MI, ^22^ 2012	prospective, multicenter, observational	412/246	NA	NA	NA	NA	NA	NA	NA	NA	NA	NA	NA	3 years
Italian Elderly ACS, ^16^ 2012	RCT, multicenter,	154/159	all-cause mortality, MI, stroke, and repeat hospital stay	81.8/81.8	49/51	92/85	44/50	38/41	49/48	10/8.9	NA	71/78	17/6	1 year
ACSIS, ^23^ 2013	prospective, multicenter, observational	192/158	NA	83	54	81	NA	36	NA	29	NA	NA	6	1 year
PL-ACS, ^24^ 2013	prospective, multicenter, observational	3288/10419	NA	82/83	47.5/37.2	78.6/71.0	36.3/32.6	30.9/30.5	NA	NA	NA	NA	4.6/0.1	2 years
Kolte, ^25^ 2013	retrospective, multicenter, observational	161640/ 806902	NA	83.9/86.2	48.2/41	72/62.5	51.9/33.7	28.0/29.1	NA	34.8/55	16.8/22.8	NA	NA	in-hospital
MONICA/KORA MI, ^26^ 2016	prospective, multicenter, observational	360/286	NA	78/80	55.8/46/5	90.6/85.0	47.8/33.6	40.0/50.0	NA	NA	24.7/36.4	NA	24.7/4.9	28 days
Conti, ^27^ 2016	prospective, single-center, observational	301/152	death and MI	80	52.8/34.2	91.7/90.8	53.5/38.8	41.2/42.1	NA	NA	NA	NA	NA	1 year
HUMIR, ^28^ 2016	prospective, multicenter, observational	654	NA	NA	NA	NA	NA	NA	NA	NA	NA	NA	NA	1 year
After Eighty study, ^17^ 2016	RCT, multicenter	229/228	MI, need for urgent revascularisation, stroke, and death	84.7/84.9	55/44	57/61	NA	20/14	NA	NA	NA	90	NA	3 years
AMI-OPTIMA, ^40^ 2018	sub-analysis of RCT, multicenter	548/610	NA	85.6	47.6	78.3	53.4	31.2	NA	NA	25.3	NA	NA	in-hospital
Liu, ^29^ 2018	retrospective, single-center, observational	319	NA	NA	NA	NA	NA	NA	NA	NA	NA	NA	NA	32,3 months
LONGEVO-SCA, ^30^ 2018	prospective, multicenter, observational	407/124	cardiac death, MI, or need for unplanned coronary revascularization	83.6/86.7	64.7/50.0	84.0/87.1	NA	39.0/42.7	53.2/53.7	14.3/29.0	NA	84.5	NA	6 months
SWEDEHEART, ^31^ 2018	prospective, multicenter, observational	4158/9696	NA	84/86	57/46	63/63	NA	NA	NA	NA	NA	NA	NA	5 years
Kvakkestad, ^32^ 2019	prospective, single center, observational	1200/864	NA	80.4/86.4	60.6/39.7	50.1/48.4	NA	20.7/19.0	NA	NA	NA	NA	NA	7 years
CAMI, ^33^ 2019	prospective, multicenter, observational	551/1900	all-cause death, MI, and stroke	80.37	54.6	65.8	6.0	25.0	53.41	10.8	NA	NA	10.8	in-hospital
Sui, ^34^ 2019	retrospective, single-center, observational	139/93	NA	83.4/84.8	52.5/57.0	66.9/68.8	93.5/90.3	40.3/34.4	51.9/45.3	NA	23.7/36.6	NA	NA	4 years
Gonçalves, ^35^ 2020	retrospective, multicenter, observational	237/87	NA	87/87	54.0/60.9	86.4/90.7	58.8/59.5	29.6/43.7	52/49	NA	14.1/9.4	64.5/80.7	NA	1 year
Hirlekar, ^18^ 2020	RCT, multicenter	93/93	MI, urgent revascularization, all-cause mortality, stroke, and recurrent hospitalization	84/84	50.5/59.1	59.1/63.4	22.6/17.2	17.2/21.5	55/54	10.8/8.6	63.4/75.3	NA	NA	1 year
SENIOR-NSTEMI, ^36^ 2020	prospective, multicenter, observational	655/845	NA	85.3/86.9	60/50	62/54	42/30	26/24	NA	17/24	7/8	NA	NA	4,8 years
Nguyen, ^37^ 2020	prospective, multicenter, observational	42/78	all-cause mortality, MI, stroke	84.1/85.2	59.5/44.9	97.6/85.9	NA	21.4/23.1	NA	21.4/48.7	NA	NA	NA	3 months
Phan, ^38^ 2020	retrospective, single-center, observational	890/543	NA	83.3/83.3	64.6/67.4	91.9/94.1	93.0/93.6	49.1/56.9	51/45	44.5/64.8	58.1/63.9	NA	NA	2,6 years
Kunniardy, ^39^ 2021	retrospective, single- center, observational	99/953	NA	NA	NA	NA	NA	NA	NA	NA	NA	NA	NA	1,3 years

All studies adopted 5% of statistical significance.Data were reported as invasive/conservative management.CABG: coronary artery bypass grafting; MACE: major adverse cardiovascular events; LVEF: left ventricular ejection fraction; MI: myocardial infarction; NA: not avaliable; PCI: percutaneous coronary intervention; RCT: randomized controlled trial.

### Clinical outcomes based on RCTs

It was obvious that invasive management was associated with lower risks of MI (OR: 0.51; 95% CI: 0.40-0.65; I^2^=0%; Figure 2B), without significant influences on all-cause death (Figure 2A), stroke (Figure 2C) or major bleeding (Figure 2D). For all-cause death (Figure 3A), the cumulative Z-curve did not cross the conventional statistical boundaries, the trial sequential monitoring boundaries, or the diversity-adjusted required information size, indicating that sufficient information was not obtained. The cumulative Z-curve crossed the trial sequential monitoring boundaries for benefit in MI (Figure 3B), indicating sufficient information was obtained. For stroke (Figure 3C) and major bleeding (Figure 3D), TSA results were ignored due to too little information used.

**Figure 2:** – Comparisons of Primary Outcomes Based on Randomized Controlled Trials. A) all-cause death; B) myocardial infarction; C) stroke; D) major bleeding.

**Figure 3:** – TSA Results for Primary Outcomes. A) all-cause death; B) myocardial infarction; C) stroke; D) major bleeding. TSA: trial sequential analysis. The blue line represents the cumulative Z-score of the meta-analysis. The red transverse lines represent the conventional statistical boundaries of p = 0.05. The red inward-sloping lines represent the trial sequential monitoring boundaries. The red outward sloping lines represent the futility boundary. The red vertical lines represent the diversity-adjusted required information size.

As to secondary outcomes, invasive management was associated with lower risks of MACE (Supplementary material 1A) and revascularization (Supplementary material 1C) compared with conservative management, without significant differences in cardiac death (Supplementary material 1B) or re-admission (Supplementary material 1D). Cumulative Z-curve crossed the trial sequential monitoring boundaries for benefit in MACE (Supplementary material 2A) and revascularization (Supplementary material 2C). In contrast, sufficient information was not obtained for cardiac death (Supplementary material 2B) or re-admission (Supplementary material 2D).

### Pooling results from RCTs and observational studies with multivariable adjustment

As TSA results revealed that sufficient information was only obtained for MI, MACE, and revascularization but not for other outcomes, results from RCTs and observational studies with multivariable adjustment were also calculated to enlarge sample size and mitigate bias as much as possible. The results indicated that invasive management was consistently associated with lower risks of all-cause death (OR: 0.57; 95% CI: 0.50-0.64; I^2^=86.4%; Figure 4A), MI (OR: 0.63; 95% CI: 0.56-0.71; I^2^=0%; Figure 4B), and stroke (OR: 0.59; 95% CI: 0.51-0.69; I^2^=0%; Figure 4C) relative to conservative management, without increasing the risk of major bleeding (Figure 4D). Additionally, invasive management could reduce the risk of MACE (Supplementary material 3A) without significant influences on cardiac death (Supplementary material 3B), revascularization (Supplementary material 3C), or re-admission (Supplementary material 3D).

**Figure 4:** – Pooling Primary Outcomes from Randomized Controlled Trials and Observational Studies with Multivariable Adjustment. A) all-cause death; B) myocardial infarction; C) stroke; D) major bleeding.

### The real-world scenario based on observational studies

Results from observational studies revealed that invasive management might decrease the risks of all-cause death (OR: 0.35; 95% CI: 0.28-0.44; I^2^=96.7%; Figure 5A) and stroke (OR: 0.47; 95% CI: 0.36-0.60; I^2^=26.0%; Figure 5C), without impact on MI (Figure 5B) and major bleeding (Figure 5D). Additionally, invasive management may decrease the risks of MACE (OR: 0.41; 95% CI: 0.32-0.53; I^2^=64.2%;Supplementary material 4A) and cardiac death (OR: 0.32; 95% CI: 0.23-0.47; I^2^=0%;Supplementary material 4B).

**Figure 5:** – Comparisons of Primary Outcomes Based on Real-World Observational Studies. A) all-cause death; B) myocardial infarction; C) stroke; D) major bleeding.

### Publication bias, sensitivity analyses, meta-regression analyses, and subgroup analyses

Funnel-plot distributions (Supplementary material 5) and Begg’s tests (Supplementary material 6) revealed no publication bias for all outcomes. In leave-one-out sensitivity analyses, the results remained consistent with the primary analysis (Supplementary material 7). Meta-regression analyses on age and the percentage of invasive management revealed no effects on clinical outcomes between invasive and conservative management (Supplementary Table 1). Subgroup analysis of all-cause death according to patients’ age suggested that the benefits in all-cause death (Supplementary material 8A), MI (Supplementary material 8B), and stroke (Supplementary material 8C) were consistent except for older patients aged more than 85 years, in whom the invasive management may increase the risk of major bleeding (OR: 2.68; 95% CI: 1.12-6.42; I^2^=0%;Supplementary material 8D) with no benefits in other parameters evaluated. For secondary outcomes, invasive management was associated with a lower risk of MACE (Supplementary material 9A) regardless of age, with reduced risk of cardiac death (Supplementary material 9B) and revascularization (Supplementary material 9C) in patients aged more than 80 years.

## Discussion

In this meta-analysis and TSA, our findings can be summarized as follows: (1) invasive management decreased the risks of MI, MACE, and revascularization with firm evidence based on RCTs and TSA results; (2) no significant differences in all-cause death, stroke, major bleeding, cardiac death, and re-admission between invasive and conservative management in RCTs may be explained by the limited number of included patients according to TSA results; (3) pooling results from RCTs and observational studies with multivariable adjustment revealed that invasive management was associated with lower risks of all-cause death, MI, stroke, and MACE relative to conservative management; (4) real-world scenario from observational studies also suggested that invasive management may decrease the risks of all-cause death, stroke, MACE, and cardiac death; (5) for older patients aged ≥85 years, invasive management may increase the risk of major bleeding.

For a long time, treating elderly patients with NSTEMI has been a challenging and tricky issue as these older patients are more likely than their younger counterparts to present atypical symptoms, such as an absence of chest pain in ACS.^41^Further, older age per se is considered an independent risk factor for early morbidity and mortality following the presentation of NSTEMI.^42^Additionally, the poorer outcome associated with elderly patients is influenced not only by extensive coronary artery disease but also by more complex comorbidities,^43^such as complex multivessel coronary calcification disease, tortuous vascular anatomy, impaired ventricular function, higher risk profile, and substantial comorbidity.^44^All of the reasons above have contributed to the uncertainty about the risk-benefit ratio of invasive management.

Real-world data showed that older patients with NSTEMI accompanied by multiple comorbidities were less likely to receive invasive management, possibly due to a perceived unfavorable risk-benefit ratio. Our meta-analysis shows that patients who did not receive invasive management were more likely to have heart failure or renal failure. Maybe the worries about contrast-induced nephropathy hindered them from receiving invasive management. In the After Eighty study,^17^457 NSTEMI patients aged ≥80 years were randomly assigned to an invasive strategy (n=229) or a conservative strategy (n=228). During a median follow-up of 1.53 years, the primary outcome defined as a composite of MI, need for urgent revascularization, stroke, and death occurred less frequently in the invasive group compared with the conservative group (40.6% vs. 61.4%; hazard ratio [HR]: 0.53; 95% CI: 0.41–0.69; p=0.0001), which was mainly due to the reduced risks of MI (HR: 0.52; 95% CI: 0.35–0.76; p=0.0010) and urgent revascularization (HR: 0.19; 95% CI: 0.07–0.52; p=0.0010). In the meta-analysis conducted by Abusnina et al., they compared the efficiency of an invasive strategy in NSTEMI patients aged more than 80 years. A total of three RCTs and 893 patients were included. Compared with the conservative strategy, the invasive strategy was associated with reduced risks of MI (relative risk [RR]: 0.58; 95% CI: 0.44-0.77; p=0.0002) and revascularization (RR: 0.24; 95% CI: 0.13-0.46; p<0.0001), without differences in all-cause mortality (RR: 0.89; 95% CI: 0.68-1.16; p=0.40), major bleeding (RR: 1.56; 95% CI: 0.60-4.05; p=0.36), or stroke (RR: 0.78; 95% CI: 0.39-1.956; p=0.48).^45^However, according to our TSA results, the nonsignificant results in all-cause death, stroke, and major bleeding between invasive and conservative management in RCTs may be explained by the limited number of included patients and underpowered for the outcomes of interest. Therefore, further studies are needed to validate the effect of an invasive strategy on patients with NSTEMI. Moreover, considering the limited number of RCTs, pooling results from RCTs and observational studies with multivariable adjustment were also conducted as supplementary analyses. The inclusion of the latest relevant studies may make the results more convincing. Additionally, the inclusion and exclusion criteria were strict in RCTs, which may limit the generality of the results to real-world practice; therefore, subgroup analysis just based on observational studies was also performed. All of the analyses above consistently indicated that the invasive strategy was superior to the conservative management. However, an increased risk of major bleeding was observed in patients aged ≥85 years, which suggested that caution should be paid when considering invasive management in very old patients.^46^

In real-world practice, approximately half of the NSTEMI patients with significant stenosis did not undergo PCI.^19^The reasons could be explained by small vessel disease ineligible for invasive management, severe coronary artery disease (e.g., multivessel/left main) combined with severe peripheral disease and severe coronary artery disease after CABG ineligible for redo surgery and PCI. In the study conducted by Phan et al.,^38^the two most common reasons for conservative management were reported: 1. poor candidacy for invasive management due to frailty, suboptimal coronary anatomy, medical comorbidities, or other reasons at the discretion of the physician (38.9%); 2. significant obstructive coronary artery disease with high risk-benefit ratio, which favors a trial of medical therapy first (36.3%).

Data from our meta-analysis revealed a positive association between an invasive strategy and better outcomes, yet the benefit of an invasive strategy might be diluted by the weight of age, with an increased risk of major bleeding in patients aged ≥85 years. Due to the limited number of RCTs, more extensive studies and RCTs are mandatory to clarify the role of invasive management in older patients with NSTEMI. The SENIOR-RITA trial (NCT03052036) is designed to determine whether an invasive strategy reduces cardiovascular death or MI in NSTEMI patients aged ≥75 years when compared with a conservative management strategy. However, the trial is estimated to be completed by 2029.

### Limitation

Some limitations should be acknowledged in our meta-analysis. First, invasive management was performed in mixed PCI and/or CABG manners. However, subgroup analyses based on recent RCTs demonstrated comparable outcomes after PCI or CABG in older patients, with PCI being preferred for frail patients with higher risks of periprocedural events, while CABG is better at achieving complete revascularization.^47 , 48^Second, MACE is commonly used in our included studies, but their components and combinations differ. Therefore, MACE was regarded as a secondary outcome instead of a primary one in our meta-analysis.

## Conclusion

Among older patients (≥75 years) with NSTEMI, invasive management could decrease the risks of MI, MACE, and revascularization with firm evidence based on RCTs and TSA results. Pooling results from RCTs and observational studies with multivariable adjustment consistently indicated that invasive management was better in improving prognosis. However, for very older patients aged ≥85 years, invasive management may increase the risk of major bleeding, which should raise our attention.

## *Supplemental Materials

For additional information, please, click here
